# Subtype‐Specific Detection in Stage Ia Breast Cancer: Integrating Raman Spectroscopy, Machine Learning, and Liquid Biopsy for Personalised Diagnostics

**DOI:** 10.1002/jbio.202400427

**Published:** 2024-11-25

**Authors:** Kevin Saruni Tipatet, Katie Hanna, Liam Davison‐Gates, Mario Kerst, Andrew Downes

**Affiliations:** ^1^ Institute for BioEngineering, School of Engineering University of Edinburgh Edinburgh UK; ^2^ Promotionskolleg NRW Bochum Germany; ^3^ Institute of Medical Sciences, School of Medicine, Medical Sciences and Nutrition University of Aberdeen Aberdeen UK; ^4^ Faculty of Life Sciences Rhine‐Waal University of Applied Sciences Kleve Germany

**Keywords:** breast cancer subtyping, early detection, liquid biopsy, machine learning, personalised cancer care, Raman spectroscopy

## Abstract

This study explores the integration of Raman spectroscopy (RS) with machine learning for the early detection and subtyping of breast cancer using blood plasma samples. We performed detailed spectral analyses, identifying significant spectral patterns associated with cancer biomarkers. Our findings demonstrate the potential for classifying the four major subtypes of breast cancer at stage Ia with an average sensitivity and specificity of 90% and 95%, respectively, and a cross‐validated macro‐averaged area under the curve (AUC) of 0.98. This research highlights efforts to integrate vibrational spectroscopy with machine learning, enhancing cancer diagnostics through a non‐invasive, personalised approach for early detection and monitoring disease progression. This study is the first of its kind to utilise RS and machine learning to classify the four major breast cancer subtypes at stage Ia.

## Graphical abstract

This study demonstrates the power of combining Raman spectroscopy and machine learning to detect and classify breast cancer subtypes using blood plasma. With an impressive 90% sensitivity and 95% specificity, our method offers a non‐invasive approach for early cancer detection, accurately identifying the four main subtypes at stage Ia. This innovative integration of vibrational spectroscopy with AI‐based analysis holds promise for personalised cancer diagnostics, potentially transforming how we detect and monitor cancer progression.

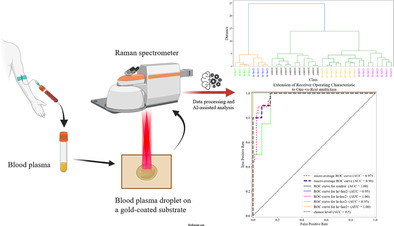

## 1 Introduction

Breast cancer (BC) in women is the most frequently diagnosed malignancy worldwide and ranks among the leading causes of cancer‐related fatalities [1]. Despite significant advancements in the prevention, diagnosis, and treatment of BC over recent decades [2], it remains a formidable global health challenge. The 5‐year survival rate for stage I breast cancer is approximately 99%, compared with 86% for stage II, 57% for stage III, and just 27% for stage IV, where the cancer has spread to distant organs [3, 4]. Often, tumours are detected only once symptoms manifest, typically at an advanced stage of the disease [5]. Distant (metastatic) recurrence is a critical clinical concern, accounting for the majority of BC‐related deaths [6]. Therefore, early detection and diagnosis while the tumour is still localised, and when treatments are most effective, are crucial for improving treatment outcomes [7].

Liquid biopsy enables the non‐invasive investigation and real‐time monitoring of a tumour's dynamic progression and response to therapy [8, 9, 10]. Liquid biopsy tests possess the potential to detect a broad spectrum of biomolecules, including circulating tumour cells (CTCs), cell‐free DNA (cfDNA), RNA, extracellular vesicles, proteins, and methylation markers, thereby providing invaluable insights into disease status. However, many current liquid biopsies aimed at early cancer detection lack the necessary sensitivity to effectively identify cancers at their earliest stages [11]. For instance, tumour‐derived genetic markers may not always be released into the bloodstream at the early stages of cancer, and when they are, their presence is often at very low levels [12]. Furthermore, cancer‐associated biomarkers routinely examined in bodily fluids may not exhibit abnormal levels even in patients with advanced cancer stages [13]. These markers also often lack specificity, as elevated levels can occur in individuals without cancer [14, 15]. Enhancing the efficacy of early cancer detection necessitates the integration of both non‐tumour derived data and direct tumour signals into the diagnostic technology [16].

Spectroscopic techniques are emerging as powerful tools in biomedical research due to their non‐invasive nature and high real‐time spatial resolution [17]. Among these techniques, Raman spectroscopy (RS) is particularly attractive because it has shown the potential to perform rapid, non‐destructive, reagent‐free, real‐time molecular analysis [18], and objective clinically relevant diagnostic information in various biomedical applications using different biofluids [19, 20, 21]. RS, a type of laser spectroscopy, excites vibrational modes within molecules at characteristic frequencies [18]. During this process, a photon loses energy when it excites a vibration, and by detecting the scattered photons with a spectrometer, we can deduce the energy loss and identify the type of vibration within the molecule. This technique reveals subtle differences in the chemical composition of cells and biological tissues, highlighting changes caused by diseases [22]. In cancer research, RS has been used in vitro to distinguish primary tumour cells from secondary tumour cells [23] and to differentiate radioresistant cells from controls [24]. It has also been applied to biopsies [25] with impressive accuracy and more recently extended to biofluids such as urine [26], tears [27], saliva [28], and blood plasma, achieving 95% accuracy for detecting stage II breast cancer [29].

The development of high‐throughput Raman instrumentation for diagnosing prostate cancer in plasma samples has been reported, demonstrating remarkable sensitivity and specificity (96.5% and 95%, respectively) in a limited patient cohort [30]. Wang et al. [31] applied spontaneous RS to analyse stage I to IV of non‐small cell lung cancer (NSCLC) and healthy samples using dried blood serum, achieving an accuracy of 65% in detecting stage I cancer from the other groups. Another study conducted measurements on whole blood samples from a group of healthy volunteers and breast cancer patients achieving classification accuracies above 94% in discriminating between control and cancer samples. However, this study focused on cancer grades 1–3, not addressing cancer stages, which provide a more comprehensive understanding of tumour progression and its impact on treatment outcomes [32]. Nargis et al. [29] employed PCA‐Factorial Discriminant Analysis (PCA‐FDA) and achieved remarkably high accuracy, with classification rates exceeding 99% for distinguishing between breast cancer patients and healthy controls. The study identified specific Raman spectral features associated with DNA and proteins that were exclusively observed in the blood plasma samples of breast cancer patients. However, it is important to note that this study was limited to patients with stages II to IV breast cancer, thereby not including earlier cancer cases from its analysis. In a subsequent study, Nargis et al. [33] conducted a comparative analysis of Surface‐Enhanced Raman Scattering (SERS) and spontaneous RS for the examination of blood serum from breast cancer patients and healthy controls using Partial Least Squares Discriminant Analysis (PLS‐DA). The results demonstrated sensitivity, specificity, and AUROC values of 90%, 98%, and 94% for SERS, and 88%, 98%, and 83% for spontaneous RS, respectively. This study was also confined to stages II to IV of breast cancer. Pereira de Souza et al. [34] applied a rapid and low‐cost ATR‐FTIR spectroscopy technique to analyse the molecular subtypes of breast cancer using blood plasma samples from patients across stages I to IV, as well as from healthy individuals. Utilising integrated Principal Component Analysis (iPCA) and Orthogonal Projections to Latent Structures Discriminant Analysis (OPLS‐DA), they achieved 100% accuracy in distinguishing the four main breast cancer subtypes. Subtype‐specific diagnosis is a major milestone towards personalised medicine [35, 36, 37, 38, 39]. However, a notable limitation of this study is that all cancer samples were pooled together, rather than being analysed according to their individual cancer stage. This pooling could mask stage‐specific spectral variations that are critical for understanding the progression and treatment of breast cancer.

We conducted a small pilot study on blood plasma from stage Ia breast cancer patients and healthy control samples, showing high sensitivity and specificity (macro‐average AUC = 0.98) in detecting different breast cancer subtypes. To the best of our knowledge, this study is the first of its kind to utilise RS and machine learning to classify the four major breast cancer subtypes: Luminal A, Luminal B, HER2‐enriched, and Triple Negative Breast Cancer (TNBC) at stage Ia. Using a custom‐built pattern recognition program, we were able to identify spectral features associated with cancer biomarkers.

## 2 Materials and Methods

### 2.1 Blood Samples

The blood plasma samples, 12 samples from breast cancer patients and 12 from healthy volunteers, utilised in this study were generously provided by the Northern Ireland biobank (Ref.: c‐13290114) and Breast Cancer Now Tissue bank (Ref.: c‐12283903). Whole blood samples were collected at diagnosis and before commencement of treatment. Upon clinical histopathological assessment, all cancer samples were identified as stage Ia and classified according to their respective tumour subtype based on the hormone‐receptor (HR) and human epidermal growth factor receptor 2 (HER2) status of tissue biopsy as shown in Table S1.

### 2.2 Sample Preparation

Blood plasma samples were double‐sealed to prevent contamination and placed in a water bath set at 37°C for 5 min for thawing. The anonymised samples were then randomly placed into a sample rack; random selection reduces bias that could occur when samples of the same group are measured consecutively. Moreover, in order to minimise metabolic activity and enzymatic degradation that could occur at room temperature, samples were placed in a cooling box with ice during the waiting times before measurements. Fifty microliters liquid droplets were individually pipetted onto a gold‐coated mirror and subsequently dried by drop‐casting.

Raman spectra were collected by the Renishaw InVia Raman spectrometer equipped with a 20× (0.4 NA) objective lens (Leica, Germany) and a 600 lines/mm diffraction grating. Using laser power (approximately 60 mW at the sample) with an excitation wavelength of 785 nm, dried blood plasma samples were exposed to illumination for 20 s for each data collection on five different regions per sample over a wavenumber range of 500–1600 cm^−1^. An excitation wavelength from the NIR region was selected for this analysis to generate high‐quality and reproducible spectra [29, 30, 33, 40].

### 2.3 Spectral Preprocessing

The preprocessing of the spectral data involved several systematic steps to ensure suitability for analysis, carried out using custom‐built Python programs.

Initially, a dataframe containing the raw spectral data were created to form an array of the raw spectra. A thresholding algorithm based on Mahalanobis Distance and Principal component (PC) analysis was applied to the raw data [41]. After mapping the original data onto the compressed domain, the score values relative to PC1 and PC2 (which represent new axes in this domain) are assessed. If the score value of a spectrum for PC1 or PC2 deviates from the respective mean by more than 2.58 times (corresponding to a 99% confidence level) the standard deviation of PC1 or PC2, that spectrum is flagged as an outlier.

Baseline correction was then performed on the raw array using the Rolling Ball technique [42], with parameter 7 and a ball height of 21 and ball width of 63. Both the corrected array and the baseline were retained for further processing.

Next, the data were mean‐centred by subtracting the mean spectrum from each individual spectrum. This was followed by aligning the spectra to the *x*‐axis to maintain consistent dimensions. Subsequently, the mean‐centred data were standardised by dividing each spectrum by the standard deviation of the mean‐centred data.

To reduce noise, despiking was performed on the normalised spectra using the ‘removeCRSFast’ function with threshold parameters set to 4. Finally, the spectra were smoothed using the Whittaker method [43], with a lambda value of 10 000.

### 2.4 Analysis Using Machine Learning

Following spectral preprocessing, the data were divided into training and validation sets with a 70:30 ratio. The training dataset underwent PCA fitting and subsequent LDA transformation. Leave‐One‐Out Cross‐Validation (LOOCV) was applied to the LDA‐transformed training data. In this process, a model is trained on the entire training set except for one piece of data, which is used as the test data. The performance of the model, specifically its accuracy and *F*1 score, was evaluated in each iteration, and the model was preserved. For the validation set, PCA fitting was executed using the original eigenvectors derived from the training dataset rather than retraining with the validation dataset, aiming to reduce overfitting risks. Each leave‐one‐out iteration within the validation set was assessed against every model saved during the training phase. Performance metrics for each model were evaluated at every iteration, and the variability in these metrics across the models was quantified using the standard deviation.

### 2.5 Analysis of Spectral Features

An algorithm was developed to analyse differences in band intensities between diseased and control Raman spectra systematically. Initially, the mean spectrum for the control group was subtracted from each individual disease spectrum. This subtraction process highlighted the spectral features that differed between the two spectra, isolating potential disease‐associated changes.

To confirm the statistical significance of the annotated spectral differences, the Mann–Whitney–Wilcoxon test was applied. This non‐parametric test is suitable for comparing differences between two independent groups without assuming a normal distribution of data. The *p*‐values were calculated and displayed for each significant spectral difference as shown in Figures S3–S6.

The developed algorithm applied this method to each spectral feature (wavenumber) to test for statistical significance, allowing for the identification of specific spectral features with significant differences between the disease and control groups. This process provided insights into the biochemical alterations associated with the disease.

### 2.6 Hierarchical Clustering Analysis

Hierarchical clustering was performed on the LDA‐transformed data using the complete linkage method [44]. This method measures the distance between clusters as the maximum distance between points in the two clusters. The complete linkage method was chosen due to its tendency to create more compact clusters. The hierarchical clustering results were visualised using a dendrogram. The dendrogram displays the arrangement of the clusters formed at various levels of similarity.

## 3 Results

Figure S1 presents the difference spectra for control versus the four breast cancer subtypes Luminal A (Hr+/Her2−), Luminal B (Hr+/Her2+), HER2 enriched (Hr−/Her2+), and TNBC (Hr−/Her2−), highlighting key spectral changes associated with each disease subtype. The observed spectral differences at multiple wavenumbers suggest differences in lipids, protein and amino acid composition, essential for identifying disease‐specific biomarkers.

PCA was performed on the preprocessed Raman spectral data. To ensure a robust yet efficient analysis, we selected five principal components (PCs) that together capture approximately 50% of the total variance, as illustrated in Figure S2. This threshold was strategically chosen: it is sufficiently high to encompass the significant spectral features primarily represented in the lower PCs, while also being low enough to mitigate the risk of overfitting by excluding the less relevant features often found in higher PCs.

The statistical significance of the annotated spectral differences was validated using the Mann–Whitney–Wilcoxon test, as depicted in Figures S3–S6. Statistically significant spectral features exhibited notable differences at specific wavenumbers.

In HER2+ subtypes, as illustrated in Tables S3 and S5, common decreases were observed at 1158 cm^−1^ (C—C/C—N stretching in proteins), 1448 cm^−1^ (CH_2_ bending in lipids and proteins), and 1518 cm^−1^ (aromatic ring stretching in proteins). Conversely, in HER2− subtypes, common significant increases were identified at 1518 cm^−1^ (aromatic ring stretching in proteins), with decreases at 941 cm^−1^ (C—C stretching in proteins) and 1448 cm^−1^ (CH_2_ bending in lipids and proteins).

For HR+ subtypes, there were no significant common increases, but common decreases were noted at 941 cm^−1^ (C—C stretching in proteins) and 1448 cm^−1^ (CH_2_ bending in lipids and proteins). Detailed descriptions of which peaks increase or decrease, indicating a respective increase or reduction in the concentration of specific chemical species, can be found in Tables S2–S5.

**FIGURE 1:**
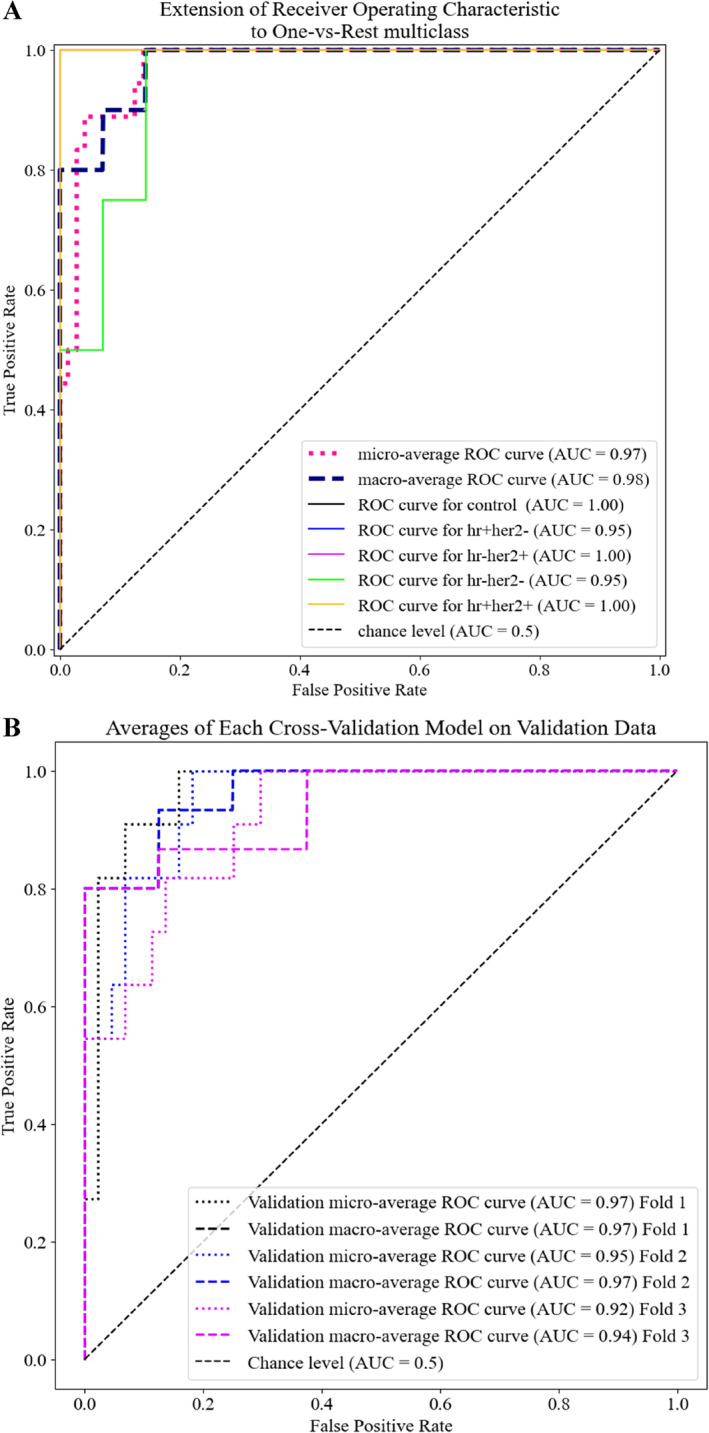
(A) Extension of the ROC analysis for control and the four breast cancer subgroups. (B) Performance of each cross‐validation model on the validation data.

The efficacy and validation of a multiclass classification model applying Receiver Operating Characteristic (ROC) analysis is illustrated. Figure 1A extends the ROC curve to a one‐vs‐rest multiclass analysis, showing the micro‐average and macro‐average ROC curves, each yielding an Area Under the Curve (AUC) of 0.97 and 0.98, respectively. Micro‐averaged ROC tends to perform well for balanced datasets, as it aggregates the contributions of all classes into a single ROC curve, often dominated by the majority class. In contrast, macro‐averaged ROC gives equal weight to each class, making it more suitable for imbalanced datasets where some classes may be underrepresented. This method ensures that the performance across minority classes is adequately captured and evaluated. In our study, the control class outweighs the four disease classes. Thus, the use of macro‐averaging is critical for properly assessing performance across all classes, especially the smaller ones.

Figure 1A also presents the ROC curves for control and the breast cancer subtypes (HR+HER2−, HR−HER2+, HR−HER2−, and HR+HER2+), all achieving an AUC of 1.00, 0.95, 1.00, 0.95, and 1.00, respectively. This indicates a high separation accuracy. Moreover, using a threshold with a relatively high true positive and low false positive rate, this model achieved 100%, 90%, 100%, 90%, and 100% sensitivity and 100%, 85%, 100%, 85%, and 100% specificity for control, HR+HER2−, HR−HER2+, HR−HER2−, and HR+HER2+, respectively.

Figure 1B illustrates the validation performance of each cross‐validation model on the validation data. The micro‐average and macro‐average ROC curves for the validation data also demonstrate high AUC values, indicating that the model maintains superior performance even on unseen validation data, thereby underscoring its robustness and generalisability.

**FIGURE 2:**
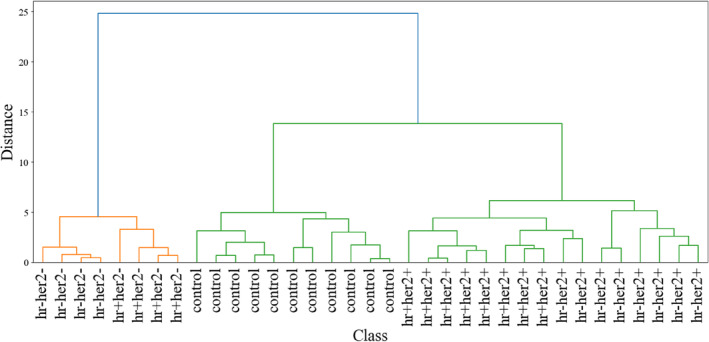
Hierarchical clustering dendrogram illustrating the relationships among blood plasma of different breast cancer subtypes and control samples of trial 1 based on their spectral features.

To elucidate the spectral features among the different breast cancer subtypes and understand their interrelationships, a hierarchical cluster analysis was conducted, as depicted in Figure 2. The dendrogram illustrates distinct clusters corresponding to the different conditions, demonstrating the model's capacity to differentiate based on spectral features.

The clustering clearly shows that samples from the same condition tend to group together, forming distinct clusters. HER2− subtypes appear to form a distinct cluster separate from the control and HER2+ subtypes, while the HER2+ subtypes also cluster separately but closer to the control samples.

**FIGURE 3:**
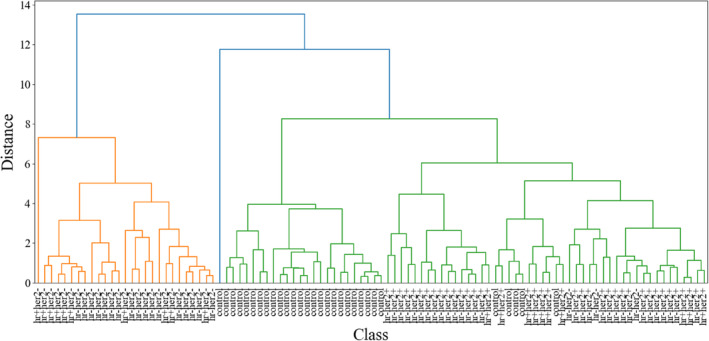
Hierarchical clustering dendrogram illustrating the relationships among blood plasma of different breast cancer subtypes and control samples of trial 2 based on their spectral features.

To validate these findings, a second trial was conducted on a different day, including additional samples from different patients. The dendrogram from the hierarchical clustering of the second trial, shown in Figure 3, indicates a similar clustering pattern as observed in the first trial.

This consistency reinforces the reliability of the initial results and demonstrates the reproducibility of the hierarchical clustering analysis.

## 4 Discussion

Our study is the first to utilise RS and machine learning to classify healthy volunteer samples alongside the four major breast cancer subtypes—Luminal A (HR+HER2−), Luminal B (HR+HER2+), HER2‐enriched (HR−HER2+), and TNBC (HR−HER2−)—at Stage Ia. The results demonstrate percentage sensitivities/specificities of 100/100 for the control group, 90/85 for Luminal A, 100/100 for Luminal B, 90/85 for TNBC, and 98/85 for HER2‐enriched. We identified key spectral features linked to cancer biomarkers, which were corroborated by multiple peer‐reviewed studies, affirming the potential of RS and machine learning in early‐stage breast cancer subtyping.

The observed intensity changes of Raman bands in this study offer significant insights into the biochemical alterations associated with cancer, supporting findings from various published studies. For instance, alterations to the 878 cm^−1^ band, associated with proline and hydroxyproline in collagen, highlights alterations in extracellular matrix components, corroborating findings by Movasaghi, Rehman, and Rehman [22].

Two comprehensive studies by Talari et al. [45] and Movasaghi, Rehman, and Rehman [22] prepared a database of molecular fingerprints of biological tissues from various studies, identifying significant spectral features linked to amide I (collagen), alanine, glycine, proline, tyrosine, amide II, amide III, and phenylalanine. These align with our observations, where significant spectral changes were identified at 643 cm^−1^ (tyrosine and phenylalanine), 861 cm^−1^ (proline and tyrosine in collagen), and 1449 cm^−1^ (amide II). These changes correspond to specific biomolecules, crucial for the structural integrity and biochemical processes within cancerous tissues.

The spectral changes observed at 861 cm^−1^, related to proline and tyrosine in collagen, indicate extracellular matrix modifications, a common feature in tumour progression, supported by studies on collagen changes in breast cancer [22]. Further support comes from two studies by Nargis et al. [29, 33], which utilised a 785 nm laser to analyse blood serum from breast cancer patients and healthy volunteers. They observed significant spectral differences at wavenumbers similar to those in our study, associated with the C—S stretching and C—C twisting of proteins/tyrosine (640 cm^−1^), collagen type I (857 cm^−1^), and lipids (1449 cm^−1^).

Notable changes in the intensity of Raman peaks when comparing breast cancer samples to normal tissue include the peak at approximately 1158 cm^−1^, linked to C—C stretching mode in β‐carotene, the peak around 1448 cm^−1^ associated with CH_2_ bending modes in lipids and proteins, the peak at approximately 1518 cm^−1^ corresponding to C=C stretching mode in β‐carotene, and peaks associated with the Amide III band in proteins. This is corroborated by Pichardo‐Molina et al. [46], who conducted a study using RS and multivariate analysis on serum samples from 11 breast cancer patients (stages II to IV) and 12 healthy controls, noting significant spectral features at 1158 cm^−1^ (beta carotene, C—C skeletal stretch), 1448 cm^−1^ (β sheet and phospholipids), and around 1518 cm^−1^ (β carotene). Medipally et al. [30] using a 785 nm laser, observed significant spectral differences at wavenumbers similar to ours, which were also attributed to β‐carotene. These bands were notably more prominent in the blood plasma of healthy volunteers compared with that of prostate cancer patients, indicating a higher expression of β‐carotene in healthy individuals. This underscores the importance of spectral features associated with β‐carotene in cancer diagnosis using RS and machine learning. Conversely, Li et al. [47] examined 1022 serum blood samples from various cancer patients (stomach, lung, liver, rectum, and oesophagus), concluding that β‐carotene concentration was lower in the serum of cancer patients compared with controls. This was similarly observed in plasma in the current study and significant differences in β‐carotene‐related peaks between control and breast cancer groups at 1158 and 1518 cm^−1^ were observed.

Cameron et al. identified the Amide II band as the most crucial wavenumber region for distinguishing between cancerous and non‐cancerous conditions in blood serum. This prominent peak captures overlapping bands associated with protein secondary structures, including α‐helices and β‐sheets. Variations in this region, as well as the Amide I region, are indicative of disease states. The Amide II band specifically reflects N—H bending and C—N stretching vibrations within protein molecules. This underscores the significance of our findings at 1448 cm^−1^, indicating that alterations in protein secondary structures are crucial for understanding the biochemical environment of cancer [48, 49]. These changes in the Amide II band are pivotal in cancer diagnostics, confirming their importance in identifying cancer‐related biochemical alterations [50].

The peak at 643 cm^−1^ is associated with tyrosine and phenylalanine, key components in protein structures, indicative of cancerous changes [22, 51]. The band at 757 cm^−1^, related to tryptophan, can provide information related to the involvement of various metabolic processes within cancer cells. At 861 cm^−1^, changes in proline and tyrosine within collagen highlight modifications in the extracellular matrix, typical of cancer [52].

Minor variations in observed wavenumbers between studies are common in RS and can arise from slight differences in instrument calibration, laser excitation wavelength, and data processing methods [53, 54]. Additionally, variations in sample conditions can slightly alter molecular vibrations, resulting in minor shifts in peak positions [54]. These variations underscore the importance of standardising protocols in RS to minimise discrepancies and improve the reproducibility and comparability of results across studies [54, 55].

Overall, the spectral differences between breast cancer and healthy control samples observed in our study are consistent with those reported in the literature, reinforcing the potential of RS in identifying and monitoring cancer biomarkers. The alignment of our findings with established studies validates the use of Raman spectral analysis combined with advanced machine learning techniques for understanding biochemical changes in cancer, enhancing diagnostic capabilities for early detection and monitoring of the disease.

The observed distinctive clustering and clear separation of the subtypes, highlight the importance of subtype‐specific disease classification. This finding aligns with the highly impactful work of Perou et al. [56], who developed the revolutionary PAM50 gene expression assay categorising breast cancer into intrinsic molecular subtypes using an assay that measures the expression levels of 50 genes (the PAM50) to classify breast cancers into one of five subtypes: Luminal A, Luminal B, HER2‐enriched, Basal‐like, and Normal‐like. This classification provides valuable prognostic information and helps guide treatment decisions by identifying the specific molecular characteristics of each tumour, offering a more precise, personalised classification system compared with traditional histopathological methods. Moreover this has been widely adopted in clinical practice and research, significantly contributing to our understanding of breast cancer heterogeneity and guiding therapeutic decisions [57]. The ability to identify specific molecular subtypes has led to better‐targeted therapies and improved patient outcomes [58, 59]. Despite this groundbreaking work by Perou et al., applying the PAM50 assay to blood samples presents several challenges. First, the assay was originally developed for tissue samples, where tumour‐specific gene expression is abundant and distinct. In blood samples, the concentration of CTCs or cell‐free tumour DNA is much lower, making it difficult to detect the specific gene expression signatures required for PAM50 classification. Additionally, the presence of a high background of normal blood cells can obscure the detection of cancer‐specific signals, necessitating highly sensitive and specific techniques to isolate and amplify the relevant genetic material. These technical difficulties complicate the direct application of PAM50 in liquid biopsy settings such as blood samples. It is, therefore, essential to continue developing fast and affordable techniques that can reliably measure a wider range of disease‐associated biochemical changes in biofluids.

The high AUC values emphasise the versatility and robustness of our model, suggesting its potential applicability across different sample types and conditions. Moreover, the consistency in cross‐validation and validation results highlights the reliability of our approach, reinforcing its suitability for deployment in diagnostic workflows.

Significant efforts were undertaken to mitigate overfitting through several layers of customised data processing methods. These included optimising data processing parameters using a LOOCV approach, conducting principal component analysis (PCA) with the original eigenvectors from the training dataset rather than retraining with the validation dataset, and dividing the data into independent training, testing, and validation datasets. Machine learning models were evaluated using a comprehensive range of performance metrics at each iteration (fold). Recognising data bias as a major limiting factor in most published vibrational spectroscopy and RS studies incorporating machine learning. This study aimed to address this issue through tailored overfitting prevention and detection techniques. However, future research will require a larger sample size for statistical validation of the results. Furthermore, acquiring samples from different cancer types and organs beyond those investigated in this study is planned to further test and validate our approach.

Overall, the application of RS with advanced machine learning techniques, as demonstrated in this study, holds significant promise for enhancing cancer diagnosis. The ability to accurately classify subtypes of a disease with high precision and reliability represents a substantial advancement in the field, paving the way for a more personalised, effective, and timely diagnosis of cancer.

## 5 Conclusion

The integration of RS, a form of vibrational spectroscopy, with AI‐based analysis in liquid biopsy presents significant potential for improving cancer detection and subtyping. This approach offers a valuable complement to traditional diagnostic methods, potentially leading to more efficient, cost‐effective, and personalised cancer treatment.

Our study adds to the expanding body of research demonstrating that RS, when combined with machine learning can accurately identify subtype‐specific molecular signatures of early (stage Ia) breast cancer. This is achieved through a carefully automated analysis of Raman spectral differences between healthy control and cancer samples, offering a promising approach for future cancer diagnostics and enhancing our understanding of the biochemical changes associated with the early development of cancer.

## Conflicts of Interest

The authors declare no conflicts of interest.

## Supporting information


Data S1.

## Data Availability

The data that support the findings of this study are available on request from the corresponding author. The data are not publicly available due to privacy or ethical restrictions.
