# Supplementary material for: Subtype‐Specific Detection in Stage Ia Breast Cancer: Integrating Raman Spectroscopy, Machine Learning, and Liquid Biopsy for Personalised Diagnostics
Source: J Biophotonics. 2024 Nov 25;18(1):e202400427. doi: 10.1002/jbio.202400427 (PMC11700701; doi:10.1002/jbio.202400427)
Supplement: Supplementary file 1 — Data S1. [file JBIO-18-e202400427-s001.docx]

**Subtype-Specific Detection in Stage Ia Breast Cancer: Integrating Raman Spectroscopy, Machine Learning, and Liquid Biopsy for Personalised Diagnostics**

Kevin Saruni Tipatet*^ab*^*, Katie Hanna*^c^*, Liam Davison-Gates*^a^*, Mario Kerst*^d^*, and Andrew Downes*^a^*

*a. Institute for BioEngineering, School of Engineering, University of Edinburgh, King’s Buildings, Edinburgh EH9 3DW, UK.*

*K.Saruni.Tipatet@ed.ac.uk*

*b. Promotionskolleg NRW, Konrad-Zuse-Straße 10, 44801 Bochum, Germany*

*c. Institute of Medical Sciences, School of Medicine, Medical Sciences and Nutrition, University of Aberdeen, Aberdeen AB24 2ZD, UK*

*d. Rhine-Waal University of Applied Sciences, Faculty of Life Sciences, Marie-Curie-Straße 1, 47533 Kleve, Germany*

***** *Corresponding authors*

**Supplementary Information**

**S. Table 1.** Demographics of the study participants.

|  | **Cancer**  *(n=12)* | **Healthy**  *(n=12)* | **Total**  *(n=24)* |
| --- | --- | --- | --- |
| **Age (mean)** | 60 | 41 |  |
| Min - Max | 37 - 84 | 28 - 61 | 28 - 84 |
| **Sex (F/M)** *n* | F=12 | F=12 | 24 |
| **Cancer subtype**, *n* |  |  |  |
| Luminal A (HR+; HER2−) | 3 | - | 3 |
| Luminal B (HR+; HER2+) | 3 | - | 3 |
| Her2+ (HR−; HER2+) | 3 | - | 3 |
| TNBC (HR−; HER2−) | 3 | - | 3 |

| 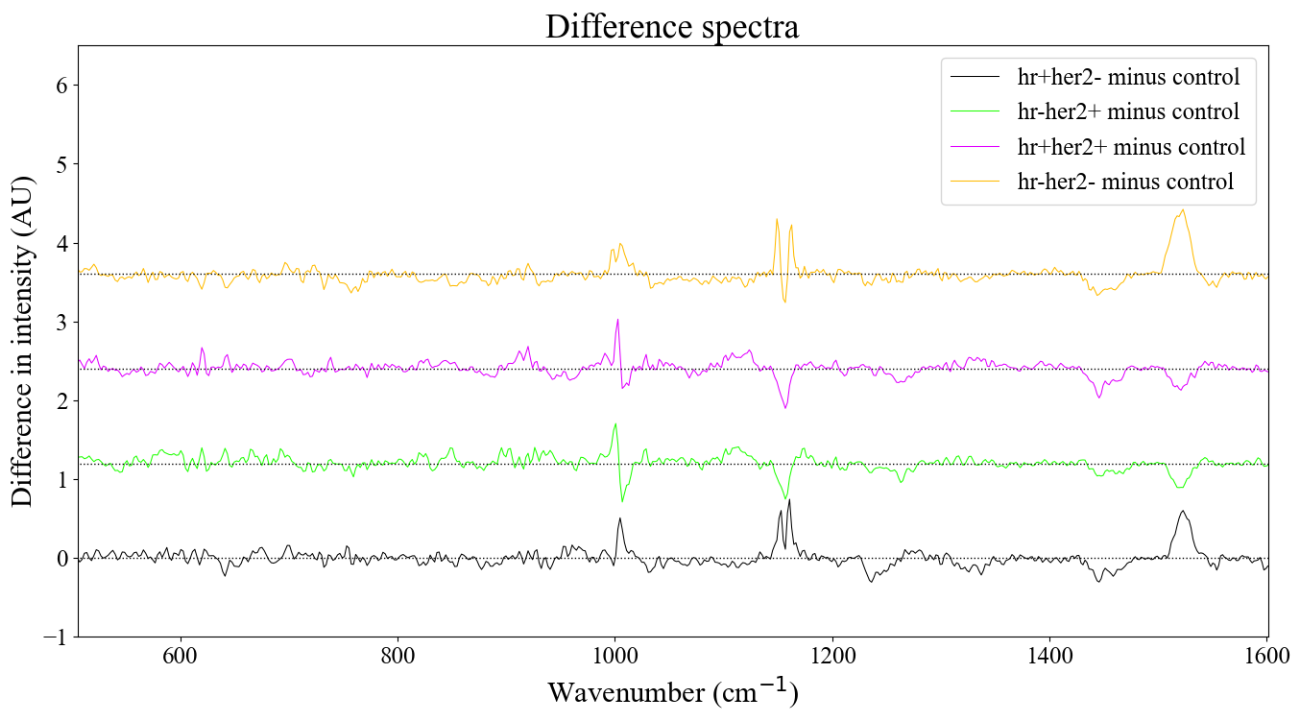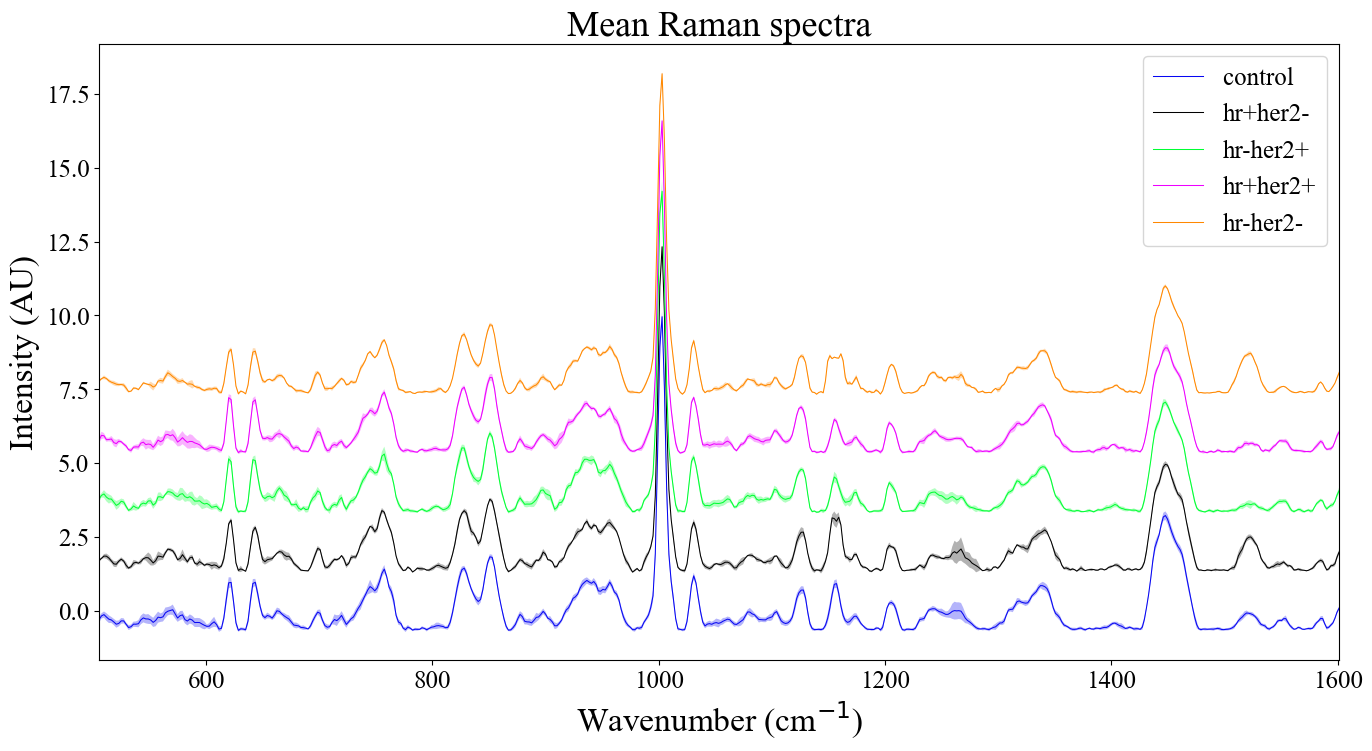 |
| --- |

**S. Fig. 1.** Mean spectra with the shaded area representing the standard deviation of the data (top). Subtype-specific difference spectra (bottom).

| 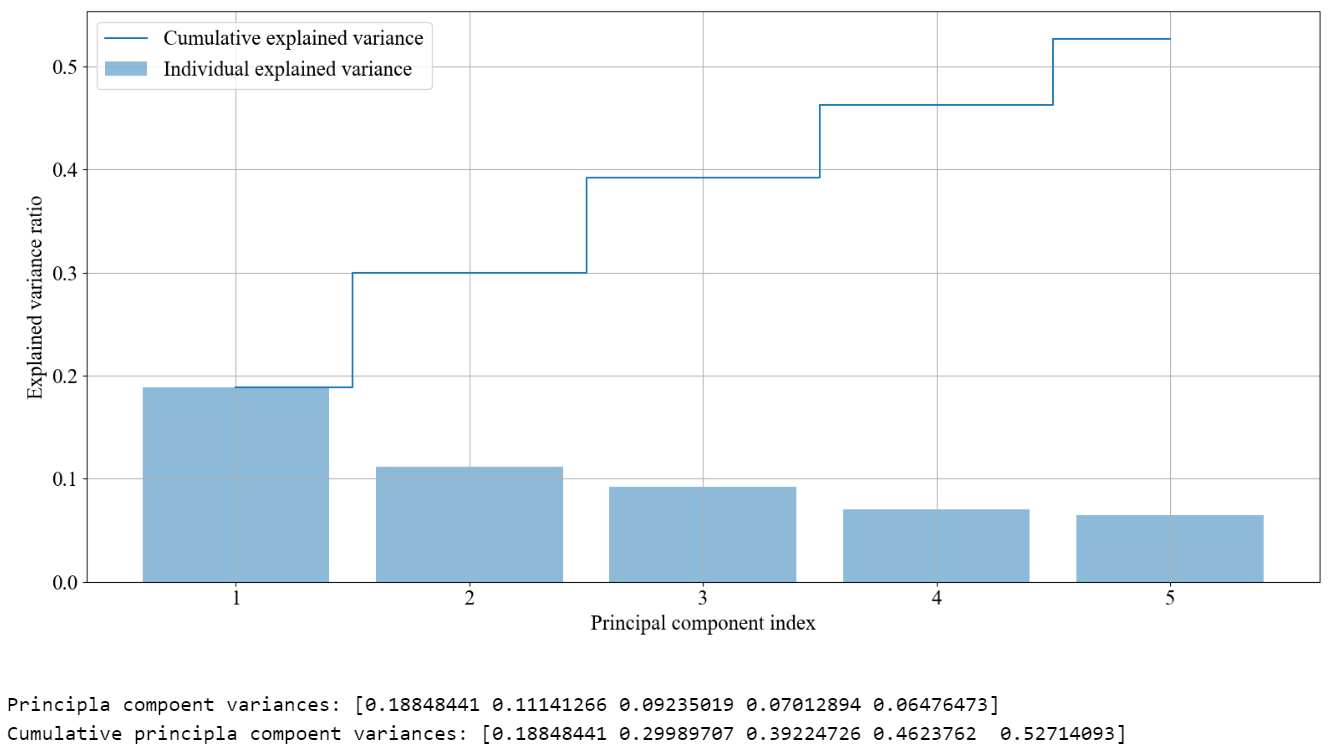 |
| --- |

**S. Fig. 2.** PCA scree plot.

| 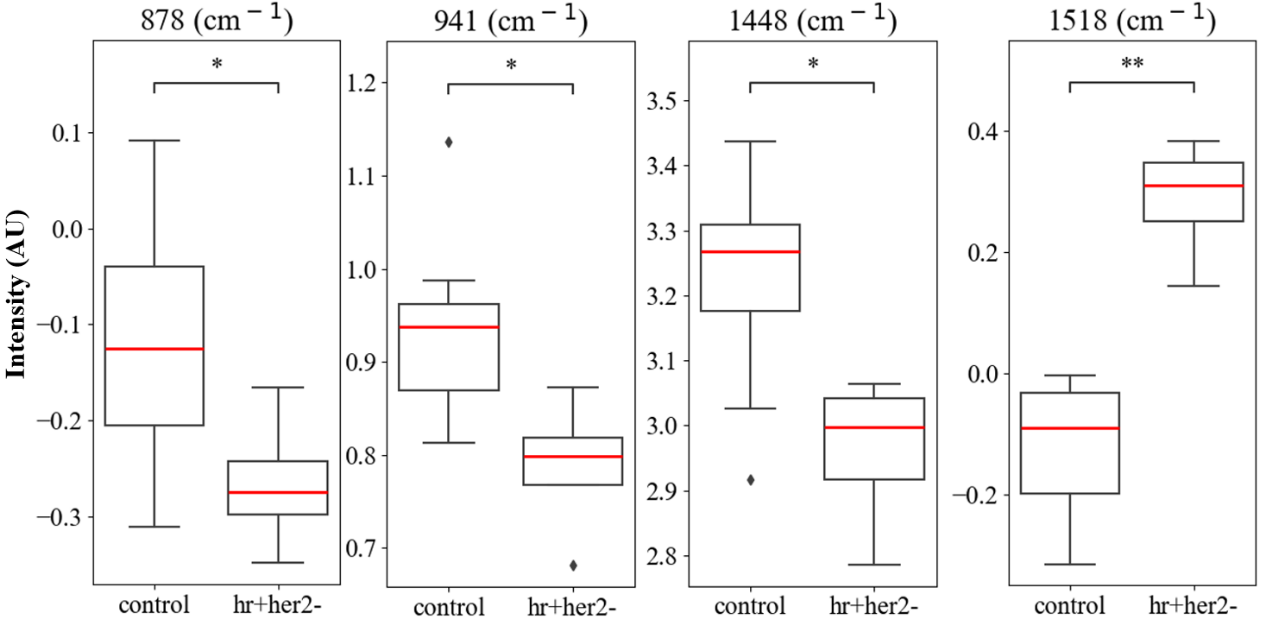 |
| --- |

**S. Fig. 3.** HR+HER2−. Spectral differences between the disease and control classes were determined by subtracting the mean control spectrum from the disease spectrum. The statistical significance of the annotated spectral differences was confirmed using the Mann-Whitney-Wilcoxon test. The p-value annotation legend is as follows: *: 1.00e-02 < p ≤ 5.00e-02, **: 1.00e-03 < p ≤ 1.00e-02, ***: 1.00e-04 < p ≤ 1.00e-03, ****: p ≤ 1.00e-04.

**S. Table 2.** HR+HER2− subtype.

| **Increase** | **Wavenumber (cm⁻¹)** | **Associated Biomarker** | **References** |
| --- | --- | --- | --- |
|  | 1518 | Aromatic ring stretching in proteins | [1-4] |
| **Decrease** | 878 | Hydroxyproline in collagen | [5] |
|  | 941 | C−C stretching (proteins) | [6] |
|  | 1448 | CH_2_ bending in lipids and proteins | [7] |

| 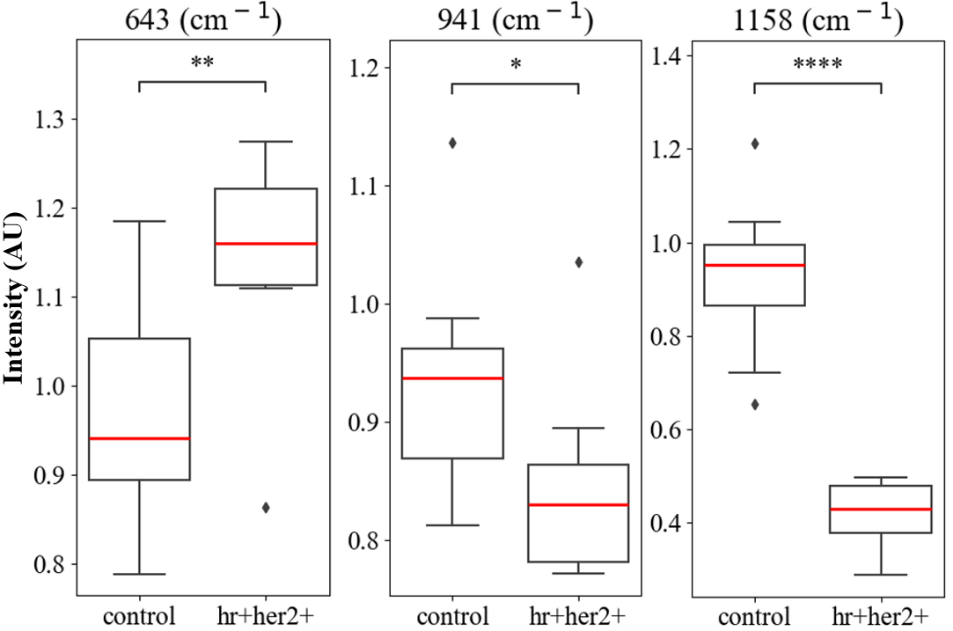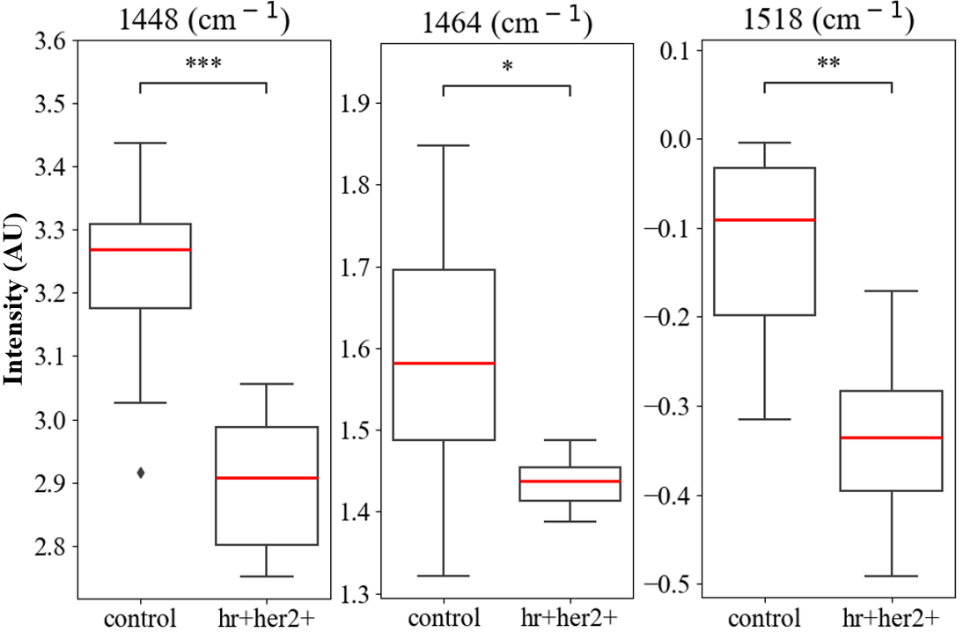 |
| --- |

**S. Fig. 4.** HR+HER2+. Spectral differences between the disease and control classes were determined by subtracting the mean control spectrum from the disease spectrum. The statistical significance of the annotated spectral differences was confirmed using the Mann-Whitney-Wilcoxon test. The p-value annotation legend is as follows: *: 1.00e-02 < p ≤ 5.00e-02, **: 1.00e-03 < p ≤ 1.00e-02, ***: 1.00e-04 < p ≤ 1.00e-03, ****: p ≤ 1.00e-04.

**S. Table 3.** HR+HER2+ subtype.

| **Increase** | **Wavenumber (cm⁻¹)** | **Associated Biomarker** | **References** |
| --- | --- | --- | --- |
|  | 643 | Tyrosine and Phenylalanine | [5, 8] |
| **Decrease** | 941 | C−C stretching (proteins) | [6] |
|  | 1158 | C−C / C−N stretching in proteins | [7] |
|  | 1448 | CH_2_ bending in lipids and proteins | [7] |
|  | 1464 | CH_2_ bending in lipids and proteins | [1, 9-15] |
|  | 1518 | Aromatic ring stretching in proteins | [1-4] |

| 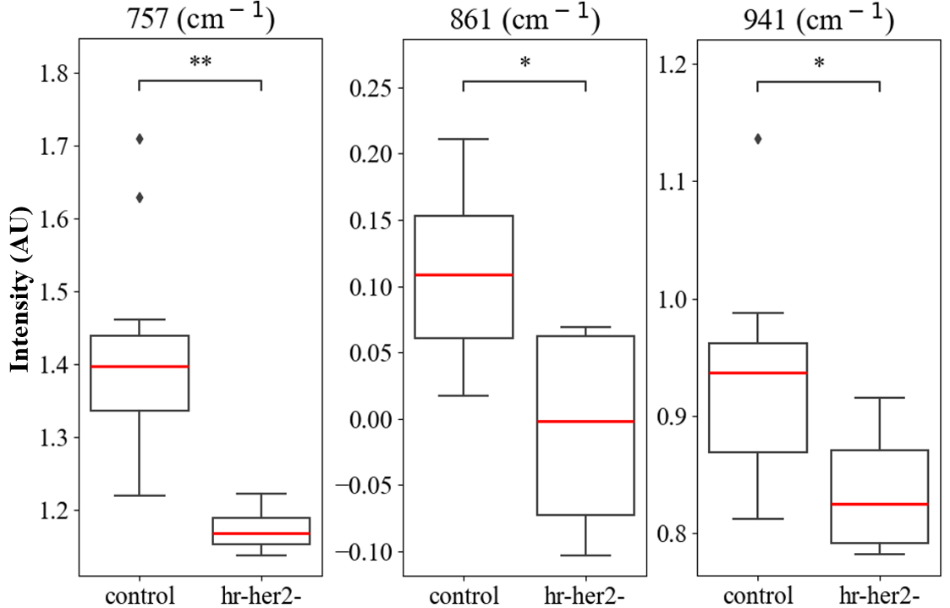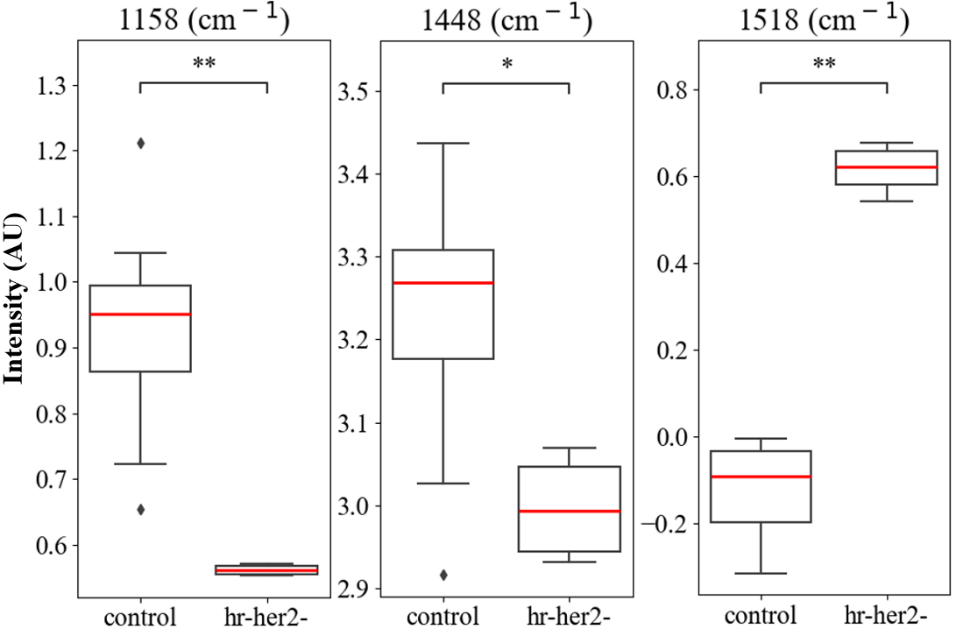 |
| --- |

**S. Fig. 5.** HR−HER2−. Spectral differences between the disease and control classes were determined by subtracting the mean control spectrum from the disease spectrum. The statistical significance of the annotated spectral differences was confirmed using the Mann-Whitney-Wilcoxon test. The p-value annotation legend is as follows: *: 1.00e-02 < p ≤ 5.00e-02, **: 1.00e-03 < p ≤ 1.00e-02, ***: 1.00e-04 < p ≤ 1.00e-03, ****: p ≤ 1.00e-04.

**S. Table 4.** HR−HER2− subtype.

| **Increase** | **Wavenumber (cm⁻¹)** | **Associated Biomarker** | **References** |
| --- | --- | --- | --- |
|  | 1518 | Aromatic ring stretching in proteins | [1-4] |
| **Decrease** | 757 | Tryptophan in proteins | [1, 9, 16, 17] |
|  | 861 | Proline and hydroxyproline | [5, 18, 19] |
|  | 941 | C−C stretching in proteins | [6] |
|  | 1158 | C−C / C−N stretching in proteins | [7] |
|  | 1448 | CH_2_ bending in lipids and proteins | [7] |

| 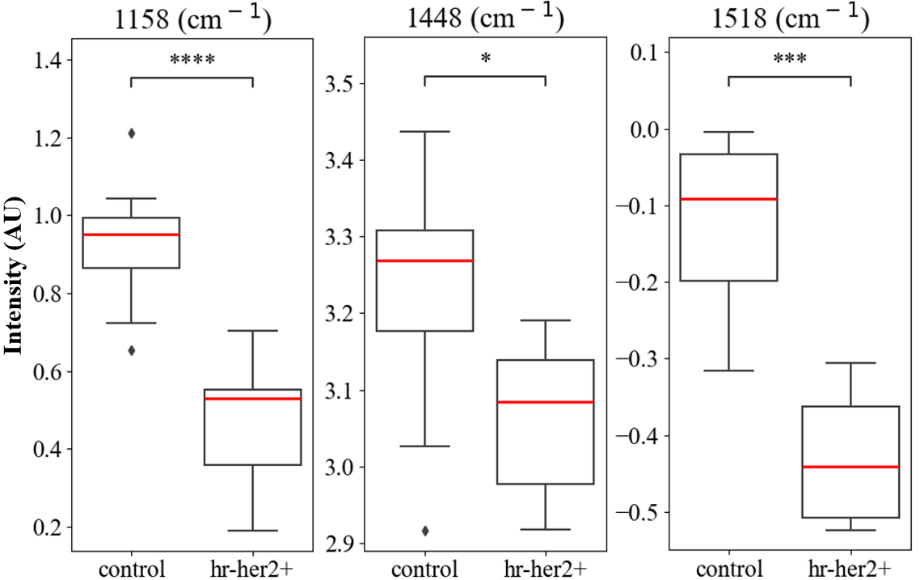 |
| --- |

**S. Fig. 6.** HR−HER2+. Spectral differences between the disease and control classes were determined by subtracting the mean control spectrum from the disease spectrum. The statistical significance of the annotated spectral differences was confirmed using the Mann-Whitney-Wilcoxon test. The p-value annotation legend is as follows: *: 1.00e-02 < p ≤ 5.00e-02, **: 1.00e-03 < p ≤ 1.00e-02, ***: 1.00e-04 < p ≤ 1.00e-03, ****: p ≤ 1.00e-04.

**S. Table 5.** HR−HER2+ subtype.

|  | **Wavenumber (cm⁻¹)** | **Associated Biomarker** | **References** |
| --- | --- | --- | --- |
| **Decrease** | 1158 | C−C / C−N stretching in proteins | [7] |
|  | 1448 | CH_2_ bending in lipids and proteins | [7] |
|  | 1518 | Aromatic ring stretching in proteins | [1-4] |

**References**

[1] A. V. Vlasov *et al.*, "Raman scattering: from structural biology to medical applications," *Crystals,* vol. 10, no. 1, p. 38, 2020.

[2] A. Mahadevan-Jansen and R. Richards-Kortum, "Raman spectroscopy for cancer detection: a review," 1997, vol. 6: IEEE, pp. 2722-2728.

[3] Z. Huang, A. McWilliams, H. Lui, D. I. McLean, S. Lam, and H. Zeng, "Near‐infrared Raman spectroscopy for optical diagnosis of lung cancer," *International journal of cancer,* vol. 107, no. 6, pp. 1047-1052, 2003.

[4] R. Malini *et al.*, "Discrimination of normal, inflammatory, premalignant, and malignant oral tissue: a Raman spectroscopy study," *Biopolymers: Original Research on Biomolecules,* vol. 81, no. 3, pp. 179-193, 2006.

[5] E. A. Klein *et al.*, "Clinical validation of a targeted methylation-based multi-cancer early detection test using an independent validation set," *Annals of Oncology,* vol. 32, no. 9, pp. 1167-1177, 2021, doi: 10.1016/j.annonc.2021.05.806.

[6] K. Hanna, E. Krzoska, A. M. Shaaban, D. Muirhead, R. Abu-Eid, and V. Speirs, "Raman spectroscopy: Current applications in breast cancer diagnosis, challenges and future prospects," *British journal of cancer,* vol. 126, no. 8, pp. 1125-1139, 2022.

[7] H. Kuper, H. O. Adami, and D. Trichopoulos, "Infections as a major preventable cause of human cancer," *Journal of internal medicine,* vol. 249, no. S741, pp. 61-74, 2001.

[8] N. M. Pereira de Souza *et al.*, "Rapid and low-cost liquid biopsy with ATR-FTIR spectroscopy to discriminate the molecular subtypes of breast cancer," *Talanta,* vol. 254, p. 123858, 2023/03/01/ 2023, doi: <https://doi.org/10.1016/j.talanta.2022.123858>.

[9] N. Kuhar, S. Sil, T. Verma, and S. Umapathy, "Challenges in application of Raman spectroscopy to biology and materials," *RSC advances,* vol. 8, no. 46, pp. 25888-25908, 2018.

[10] J. W. Chan, D. S. Taylor, T. Zwerdling, S. M. Lane, K. Ihara, and T. Huser, "Micro-Raman spectroscopy detects individual neoplastic and normal hematopoietic cells," (in eng), *Biophys J,* vol. 90, no. 2, pp. 648-56, Jan 15 2006, doi: 10.1529/biophysj.105.066761.

[11] F. Bonnier and H. J. Byrne, "Understanding the molecular information contained in principal component analysis of vibrational spectra of biological systems," (in eng), *Analyst,* vol. 137, no. 2, pp. 322-32, Jan 21 2012, doi: 10.1039/c1an15821j.

[12] K. E. Shafer‐Peltier *et al.*, "Raman microspectroscopic model of human breast tissue: implications for breast cancer diagnosis in vivo," *Journal of Raman Spectroscopy,* vol. 33, no. 7, pp. 552-563, 2002.

[13] R. Malini *et al.*, "Discrimination of normal, inflammatory, premalignant, and malignant oral tissue: a Raman spectroscopy study," (in eng), *Biopolymers,* vol. 81, no. 3, pp. 179-93, Feb 15 2006, doi: 10.1002/bip.20398.

[14] S. Kaminaka, T. Ito, H. Yamazaki, E. Kohda, and H. o. Hamaguchi, "Near‐infrared multichannel Raman spectroscopy toward real‐time in vivo cancer diagnosis," *Journal of Raman Spectroscopy,* vol. 33, no. 7, pp. 498-502, 2002.

[15] Y.-Y. Tan *et al.*, "Design of auto-classifying system and its application in Raman spectroscopy diagnosis of gastric carcinoma," 2003, vol. 3: IEEE, pp. 1360-1363.

[16] N. Stone, C. Kendall, J. Smith, P. Crow, and H. Barr, "Raman spectroscopy for identification of epithelial cancers," (in eng), *Faraday Discuss,* vol. 126, pp. 141-57; discussion 169-83, 2004, doi: 10.1039/b304992b.

[17] L. Su *et al.*, "Raman spectral properties of squamous cell carcinoma of oral tissues and cells," *Laser Physics,* vol. 22, pp. 311-316, 2012.

[18] K. Pantel and C. Alix-Panabières, "Liquid biopsy and minimal residual disease—latest advances and implications for cure," *Nature Reviews Clinical Oncology,* vol. 16, no. 7, pp. 409-424, 2019.

[19] S. Connal *et al.*, "Liquid biopsies: the future of cancer early detection," *Journal of Translational Medicine,* vol. 21, no. 1, p. 118, 2023/02/11 2023, doi: 10.1186/s12967-023-03960-8.
